# Single-cell lipidomics: protocol development for reliable cellular profiling using capillary sampling†

**DOI:** 10.1039/d5an00037h

**Published:** 2025-03-07

**Authors:** Anastasia Kontiza, Johanna von Gerichten, Matt Spick, Emily Fraser, Catia Costa, Kyle D. G. Saunders, Anthony D. Whetton, Carla F. Newman, Melanie J. Bailey

**Affiliations:** a School of Chemistry and Chemical Engineering, Faculty of Engineering and Physical Sciences, University of Surrey GU2 7XH Guildford UK m.bailey@surrey.ac.uk; b School of Health Sciences, Faculty of Health and Medical Sciences, University of Surrey GU2 7XH Guildford UK; c School of Computer Science and Electronic Engineering, Faculty of Engineering and Physical Sciences, University of Surrey GU2 7XH Guildford UK; d vHive, School of Veterinary Medicine, School of Biosciences and Medicine, University of Surrey Guildford GU2 7XH UK; e GlaxoSmithKline, Cellular Imaging and Dynamics Stevenage SG1 2NY UK

## Abstract

Single-cell lipidomics enables detailed analysis of the lipidomes of cells, but is challenged by small sample volumes, the risk of background interference and a lack of validation data. In this study, we explore the effect of different sampling variables on the lipid profiles of single pancreatic cancer cells, detected using liquid chromatography-mass spectrometry (LC-MS). We use automated and manual capillary sampling methods to isolate living single cells and evaluate different sampling media, capillary tips, aspiration volume, and temperature and humidity control. We demonstrate that automated and manual capillary sampling yield comparable lipid profiles when key parameters are controlled. Our findings highlight that appropriate blank correction, capillary tip type, and the control of aspiration volumes are all critical to preserving detection sensitivity. Conversely, choice of sampling medium does not affect lipidomics results. We also set out suggested best practices for these methodological variables, laying a foundation for robust, adaptable workflows in single-cell lipidomics for applications such as biomarker discovery and metabolic research.

## Abstract 2

Single-cell lipidomics enables detailed analysis of the lipidomes of cells, but is challenged by small sample volumes, the risk of background interference and a lack of validation data. This work explores the impact of various parameters on the lipid profile obtained from single cells.

## Graphical abstract

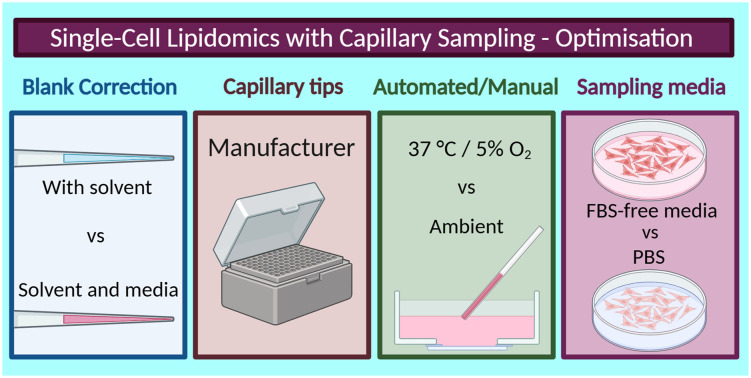

## Introduction

Single-cell analysis has shed light on cellular heterogeneity, which drives biological complexity and disease progression.^1,2^ Unlike bulk analysis, which averages measurements across cell populations, single-cell techniques attempt to expose the intricate molecular landscapes of individual cells, and to uncover unique states and functions masked in bulk measurements.^3–6^ This detail is crucial in fields like cancer research, where the behaviour of rare cell populations can significantly affect disease outcomes.^7^

Single-cell lipidomics is a nascent and rapidly-growing approach for profiling the lipid composition of individual cells in single-cell omics.^8^ Lipids are crucial for cell membrane structure, energy storage, and signalling pathways, with dysregulation linked to diseases such as metabolic disorders and cancer.^9,10^ Recent single-cell studies have shed insight into lipids shaping immune memory post infection, being able to predict colorectal cancer metastasis based on the fingerprint of circulating tumour cells, and identifying biomarkers for various breast cancer subtypes.^11–13^ Thus, single-cell lipidomics is a powerful tool for understanding cellular metabolism and identifying novel biomarkers for disease diagnosis and treatment.^14^

Mass spectrometry (MS) is the gold standard for single-cell lipidomics due to its sensitivity, specificity, and high-throughput capabilities, enabling detection of minute analytes in single cells.^15,16^ Unlike mass spectrometry imaging (MSI), which provides spatially-resolved chemical information, liquid chromatography-mass spectrometry (LC-MS) offers several distinct advantages.

Single-cell LC-MS utilises chromatographic separation before mass spectrometric detection, enhancing the detection of low-abundance lipids and providing more detailed structural information,^17^ especially compared with shotgun, or static-nanospray lipidomics, where the absence of chromatographic separation increases spectral complexity and ionisation suppression.^18–21^ Single-cell LC-MS also enables the identification of isobaric and isomeric compounds, which may be indistinguishable by MSI alone. Additionally, unlike MSI, which requires fixed or cryopreserved samples, LC-MS can be coupled with live-cell selection methods, allowing for lipid profiling from single cells collected in their natural state.^22,23^

Capillary sampling enables user-selected sampling of individual cells, providing detailed lipid profiles that reveal critical differences between cell types and states.^24–26^ This method offers several advantages over other single-cell techniques, such as laser capture microdissection (LCM) and fluorescence-activated cell sorting (FACS),^27,28^ as it allows direct extraction of living whole cells or cellular contents in their native environment with limited perturbation. Additionally, the integrated microscopy allows real-time analysis of dynamic cellular processes. Despite the lower throughput compared to other single-cell sampling techniques, capillary sampling is a versatile and valuable technique for various single-cell research applications, such as bystander effects, or the effect of stromal/neighbouring cells on developing cells. Both manual and automated capillary sampling instrumentation have been reported in literature for the sampling of single cells.^25,26,29–34^

Although capillary sampling has many advantages, the sampling process itself lacks standardisation and reproducibility, especially since a volume of cell media is aspirated alongside the cell. Previous studies do not consider this, and therefore the measured lipid profile may not faithfully represent the cell, rather the cell and its local milieu. A crucial aspect of this study is therefore the exploration of strategies for blank correction, an essential step for accurate MS-based lipidomic analyses. Proper blank correction minimises background noise and contaminants introduced during sample preparation, capillary sampling, or MS analysis.

This work employs both manual and automated capillary sampling methods to collect and analyse single pancreatic cancer (PANC-1) cells with lipidomics, with the goal of providing a data-driven analysis of best practices. We compare and evaluate methods based on lipid coverage and examine the impact of sampling under controlled (humidity and temperature) *versus* ambient conditions. Additionally, we assess the influence of varying capillary tip types, sampling media, and the volume of media aspirated with cells on lipid detection. Our findings provide new insights into protocol optimisation for single-cell lipidomics, laying a foundation for adaptable workflows that can be tailored to diverse single-cell research objectives.

## Experimental

### Cell culture

Human pancreatic adenocarcinoma cells (PANC-1, Merck, UK) were cultured in Corning® T-75 flasks (Merck, UK) using Dulbecco's modified Eagle's medium (DMEM, Sigma-Aldrich, UK, cat. no. 21969035) supplemented with 10% foetal bovine serum (FBS, Fisher Scientific, UK, cat. no. 11550356), 1% penicillin/streptomycin (Fisher Scientific, UK, cat. no. 15140122), and 2 mM l-glutamine (Sigma-Aldrich, UK, cat. no. 25030024). Cells were incubated at 37 °C under 5% CO_2_ and 21% O_2_, with the culture medium changed every two days. When cell cultures reached ∼80% confluency, cells were passaged.

For manual capillary sampling, approximately 250 000 cells were plated in BioLite™ culture dishes (Fisher Scientific, UK, cat. no. 130181), while 200 000 cells were plated in 35 mm Nunc™ Glass Bottom Dishes (Fisher Scientific, UK, cat. no. 150682) for automated sampling. In parallel, control dishes containing medium only (no cells) were also prepared to serve as negative controls. After a 72-hour incubation, the medium was removed, and cells were washed three times with warm Dulbecco's phosphate-buffered saline (PBS, Sigma-Aldrich, UK, cat. no. D8537). Cells were then maintained in either FBS-free medium or PBS for the sampling procedures.

### Manual capillary sampling

Single-cell isolation was conducted under ambient conditions, using the method introduced by Lewis *et al.*^26,35^ Capillary tips (10 μm without filament) purchased from Yokogawa and Humanix (Japan), were mounted using the tip holder on the sampling device, and cell selection was performed under a Zeiss Axiovert 40C inverted microscope. Only cells of similar size were chosen, and their diameters were verified using the AmScope software (UK). A capillary tip was fitted in a nanomanipulator (Attocube, Germany) which positioned the tip over a selected cell. A PM2000 microinjector (MicroData Instrument, USA) applied positive pressure to prevent capillary action until aspiration, which was initiated by back pressure to isolate one cell per capillary tip. Each sampled cell included a small volume of medium; additional tips were loaded with only medium from control dishes to serve as capillary blanks (see ESI Fig. 9†). Tips containing cells and blanks were placed on dry ice for the duration of the sampling process and then stored at −80 °C overnight. A typical sampling experiment to collect 40 single cells took approximately 5 hours from start to finish.

### Automated capillary sampling

Single-cell sampling using the Yokogawa SS2000 Single Cellome™ System (Japan) was conducted under incubation and humidity control (37 °C, 5% CO_2_). Single cells of comparable size were sampled using 10 μm capillary tips (Yokogawa, Japan) in direct MS mode with a pre-sampling pressure of 8 kPa, sampling pressure of 14 kPa and post-sampling pressure of 3 kPa. The puncture count was 1 and the holding time was 200 ms. Each single cell aspirated in a capillary tip carried a small volume of sampling medium and was placed in dry ice after confirmation of successful sampling. As described above, capillary blanks were also created. Following sampling with the system, capillary tips with cells and blanks were stored at −80 °C overnight. A typical sampling experiment to sample 40 single cells took approximately 4 hours from start to finish.

### Sample transfer

The capillary tips were backfilled using a 10 μL Hamilton syringe with 5 μL EquiSPLASH® (16 ng mL^−1^, Avanti Polar Lipids, cat. no. 330731) internal standard solution containing 0.01% (v/v) butylated hydroxytoluene (BHT, Fisher Scientific, UK, cat. no. 11482888) in the starting mobile phase solvent (70 : 30 A/B) of the chromatographic methods (ESI Tables 2 and 3†). The capillary tips were then fitted into a gas syringe with a Luer-lock adapter (65 μL min^−1^ flow rate), and the contents were transferred into Qsert LC-MS vials (Waters™, UK, cat. no. 186001126DV), as described previously by Saunders *et al.*^24^ Solvent blanks were created by backfilling capillary tips with mobile phase and internal standard mixture and eluting in vials. Capillary blanks were created using tips with sampling medium as described above and then backfilled with mobile phase and internal standard mixture and eluted in vials. For analytical flow LC-MS, 10 μL of the internal standard mix were added to each vial for a total volume of 15 μL per sample. The vials were then capped and stored at −80 °C until the day of LC-MS analysis.

### Lipidomics LC-MS(/MS) analysis

Lipid detection *via* LC-MS was achieved using an Ultimate 3000 UHPLC (Thermo Fisher Scientific, USA) system coupled to a Q-Exactive™ Plus Hybrid Quadrupole-Orbitrap™ mass spectrometer (Thermo Fisher Scientific, USA) with the MS method described previously by Saunders *et al.*^24^ Briefly, the heated electrospray ionisation (HESI) source temperature was set to 320 °C with 4 kV spray voltage, automatic gain control (AGC) target of 1 × 10^6^, mass range of 200–1400 *m*/*z*, and 140 000 mass resolution setting at 200 *m*/*z*. Data were acquired in positive ionisation mode, using Xcalibur (Thermo Fisher Scientific, USA). The total 15 μL sample volume was injected onto a C30 column (Accucore™, 2.6 μm, 2.1 × 150 mm, Thermo Fisher Scientific, USA) equilibrated at 40 °C with an analytical flow rate of 0.35 mL min^−1^. The solvent system (LC-MS grade, Chromasolv™ Honeywell, Fisher Scientific) was 60 : 40 (v/v) acetonitrile/water and B 85 : 10 : 5 (v/v) acetonitrile/isopropanol/water, both with 0.2% formic acid (LC-MS grade, Fisher Chemical™ Optima™, Fisher Scientific) and 10 mM ammonium formate (99%, Acros Organics). The chromatographic gradient is described in ESI Table 2.†

LC-MS/MS was also performed for single-cell lipidomics analysis, as described by von Gerichten *et al.*^25^ In brief, data-dependent analysis (DDA) was conducted using an Acquity M-class (Waters, UK) coupled to a ZenoToF 7600 mass spectrometer (SCIEX, Canada). The spray voltage was set to 4500 V, the declustering potential to 80 V, the source temperature at 350 °C, *m*/*z* range to 150–900, MS^1^ collision energy to 12 V, and collision-induced dissociation (CID) to 35 V. Data were acquired in positive ionisation mode, using Sciex OS (SCIEX, version 3.0.339). The total 5 μL sample volume was injected onto a polar C18 column (Luna Omega, 3 μm, 50 × 0.3 mm, Phenomenex, USA) equilibrated at 40 °C with a micro flow rate of 8 μL min^−1^. The solvent system was the same as described above. The gradient method is described in ESI Table 3.†

### Data analysis

MS-DIAL^36^ (Japan, version 5.4.241004) was used to process LC-MS lipidomics data (.raw files). The MS^1^ tolerance was set to 0.001 Da and the minimum peak height to 5000 with a mass slice of 0.1 Da. Adduct ions [M + H]^+^, [M + NH_4_]^+^, and [M + H–H_2_O]^+^ were allowed. For all settings, the accurate mass tolerance for MS^1^ data was set to 0.001 Da and the retention time tolerance to 0.05 min. Additionally, an in-house database of lipids previously observed in PANC-1 cells was imported. To match the expected retention times of the database to those of the LC-MS runs, a retention-time adjuster was used using the retention-time shifts observed in the internal standard lipids during the run. All scripts used to process data in this study are available on Github (https://github.com/AnastasiaKontiza/Capillary_sampling_paper). Gap filling was disabled. MS-DIAL (Japan, version 4.9.221218) was also used to process LC-MS/MS lipidomics data (.wiff files) with settings as described previously.^25^

Data were then exported to CSV files and curated using a previously-described machine learning approach to flag positive lipid identifications, based on retention time, formula, head group and polarity.^37^ Data were then analysed using Excel (Microsoft, USA), Python (version 3.9.12) and R (version 2024.09.1 + 394) programming languages.

Only peaks assigned to lipid classes represented in the internal standard (EquiSPLASH®: Cer, DAG, LPC, LPE, MG, PC, PE, PG, PI, PS, SM, TAG) and their ether form were included in the study. Blank subtraction, 3 × signal-to-background ratio filtering based on peak area, and normalisation to the internal standard were performed to report average number of lipid features. Data were log transformed and auto-scaled, with zero values replaced with half the minimum value of each sample, before multivariate statistical analysis using MetaboAnalyst 6.0.^38^ GraphPad Prism version 10.3.1 (Windows, GraphPad Software, USA) was used for creation of plots and *t*-test calculations.

**Fig. 1:**
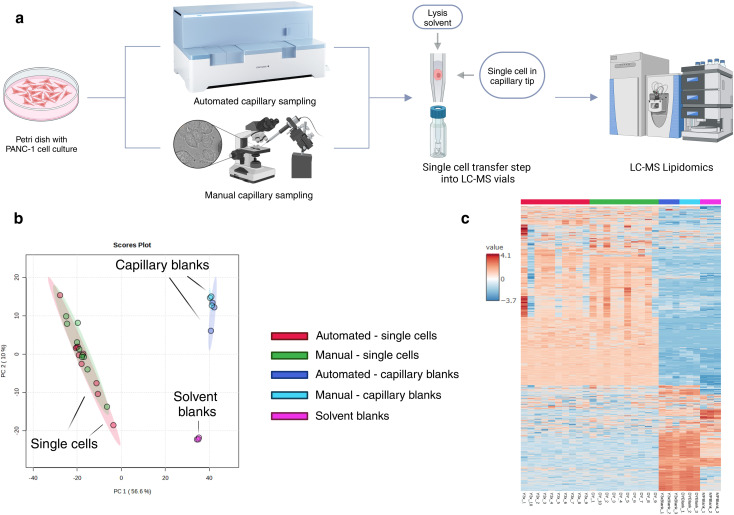
Comparison of single cells and blanks. (a) Schematic workflow of single-cell capillary sampling and downstream LC-MS analysis, created in Biorender.com, (b) PCA of the MS^1^ lipid profiles of capillary-sampled cells and blanks, (c) clustered heatmap showing MS^1^ lipids detected in single cells and corresponding blanks for automated and manual capillary sampling. Lipidomics identifications were made using a retention time and polarity-based machine learning algorithm.^37^ Data are auto-scaled and log transformed. Lipids that are present in at least 50% of single-cell groups (automated and manual capillary sampling, *n* = 10) are plotted. Solvent blanks (*n* = 3) comprised mobile phase and internal standard, while capillary blanks comprised capillary-sampled media (foetal bovine serum-free media) collected and processed in the same way as a single cell, with addition of mobile phase and internal standard, *n* = 3 for manual and automated methods.

**Fig. 2:**
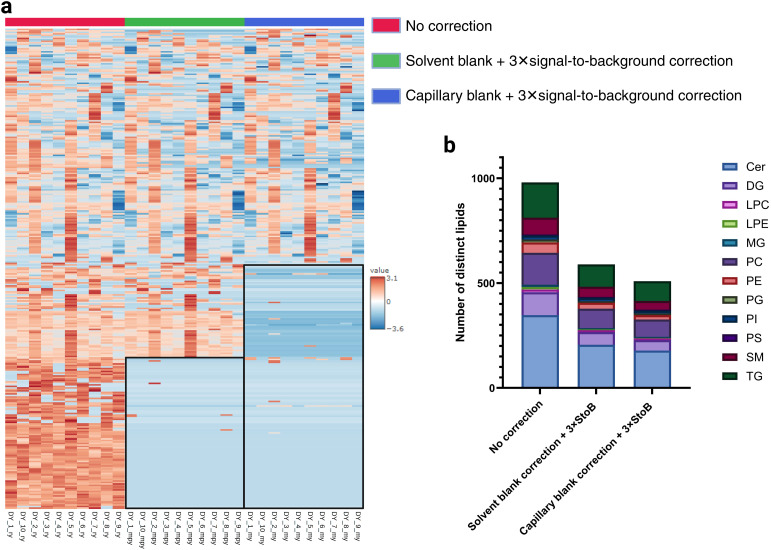
Effect of blank correction on single-cell data. (a) Clustered heatmap of lipidomics single cells collected using manual capillary sampling with Yokogawa tips; red = no blank correction, green = solvent blank and 3 × signal-to-background correction, blue = capillary blank and 3 × signal-to-background correction, (b) stacked bar chart of MS^1^ lipids identified in single cells by lipid class. From left to right, data not corrected, solvent blank and 3 × signal-to-background correction, and lastly capillary blank and 3 × signal-to-background correction. Only lipids that are present in at least 50% of single-cell groups are shown. Lipidomics identifications were made using a retention time and polarity-based machine learning algorithm.^37^

**Fig. 3:**
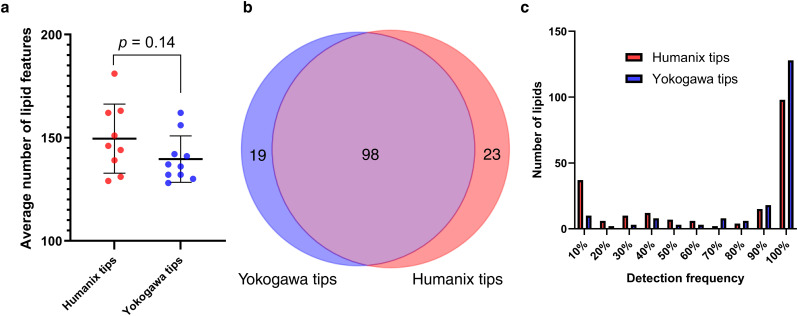
Capillary tip effect on lipid profile. (a) Average number of MS^1^ lipid features detected per single cell sampled with manual capillary sampling using Humanix (*n* = 9) and Yokogawa tips (*n* = 10). Error bars show 1 × standard deviation, *p* = 0.14, (b) Venn diagram of MS^1^ lipids detected in single cells sampled with manual capillary sampling using Humanix (*n* = 9) and Yokogawa tips (*n* = 10), plotting lipids that are found with 100% consistency in each single-cell group, (c) interleaved bar chart showing detection frequency of MS^1^ lipids detected using manual capillary sampling with Humanix and Yokogawa tips. Lipidomics identifications were made using a retention time and polarity-based machine learning algorithm,^37^ and then filtered according to an in-house lipid database.

**Fig. 4:**
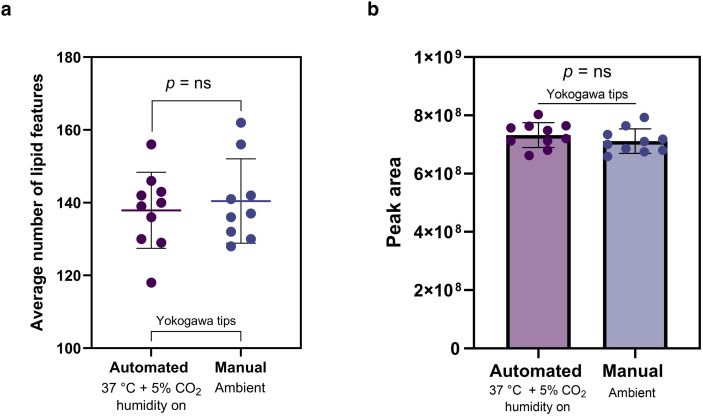
Comparison of automated and manual capillary sampling of single cells. (a) Average number of MS^1^ lipid features detected per single cell sampled in foetal bovine serum (FBS)-free media using Yokogawa tips with automated (*n* = 10) and manual (*n* = 10) capillary sampling, error bars show 1 × standard deviation, *p* = 0.62, (b) average total peak area for detected MS^1^ lipids in single cells (*n* = 10 for both automated and manual capillary sampling), *p* = 0.27, error bars show 1 × standard deviation. Lipidomics identifications were made using a retention time and polarity-based machine learning algorithm,^37^ and then filtered according to an in-house lipid database.

For Fig. 1 and 2, MS-DIAL lipidomics data outputs were verified with the retention time and polarity-based machine learning algorithm.^37^ For Fig. 3 and 4, MS-DIAL lipidomics data outputs were verified with the retention time and polarity-based machine learning algorithm, as well as filtered to include lipids only present in the in-house database of lipids previously observed in PANC-1 cells, found on GitHub (https://github.com/AnastasiaKontiza/Capillary_sampling_paper).

## Results and discussion

### Automated and manual capillary sampling for single-cell lipidomics

To determine the performance of automated and manual capillary sampling in single-cell lipidomics, we compared these methods under controlled conditions in a single batch, defining the key factors: lipid coverage, sampling medium, capillary tip type, and blank correction.

In addition to the single-cell samples created using both cell picking methods, two blank sample groups were collected.^39,40^ Specifically, *solvent blanks* comprised mobile phase and internal standard, and *capillary blanks* comprised a capillary sample from media without collection of a cell, matched to the volume of the single-cell samples by microscopy examination of the meniscus in the capillary, and later on backfilled with mobile phase and internal standard as per the sample transfer step. All blanks underwent the sample transfer, storage and analysis process as single cells (see Experimental for details).

Fig. 1a shows a schematic workflow of single-cell capillary sampling and downstream LC-MS analysis. Principal component analysis (PCA) (Fig. 1b) demonstrated a clear separation between single cells, solvent blanks, and capillary blanks for both sampling methods, confirming their effectiveness in isolating cellular lipid profiles while minimising overlap with non-cellular signals. The lipid profiles of single cells clustered closely together for both methods. Capillary blanks from both methods clustered together (anticipated, as the same FBS-free media was used in all samples). Similarly, the solvent blanks formed a distinct cluster due to the absence of media matrix components. As seen in Fig. 1c, the lipid features detected in single cells differed markedly from those in the blanks. The heatmap shows a cluster for automated and manual sampling of single cells (red and green) that differs from the cluster for the blanks (dark and light blue, and pink). Additionally, lipid features from both methods showed high similarity.

The differentiation observed in PCA highlights the ability of these methods to distinguish cellular lipids from non-cellular signals, validating their utility for single-cell analyses. Furthermore, the use of solvent blanks and capillary blanks ensured that non-cellular lipid contributions were appropriately excluded from the analyses. This differentiation underscores the necessity of robust blank correction in single-cell workflows, as it significantly enhances data specificity and reliability.

### The blank correction method impacts single-cell data

Next, we evaluated the impact of different blank correction strategies on single-cell lipid detection using capillary sampling with Yokogawa capillary tips. The clustered heatmap (Fig. 2a) displays lipids detected in at least 50% of single-cell groups across three conditions: (1) no blank correction, (2) blank correction with solvent blanks (average of 3) and 3 × signal-to-background filtering, and (3) blank correction with capillary blanks (average of 3) combined with 3 × signal-to-background filtering. An expanded version of the heatmap with the lipid names can be found in ESI Fig. 1–3.†

Fig. 2a shows that as stricter blank correction methods were applied, the number of detectable lipids decreased. The same effect can also be observed in Fig. 2b, which shows the distribution of lipids by class before any correction and with subsequent levels of blank correction. These data show that the blank correction has a considerable impact on the profile of detectable lipids.

By comparing levels of blank correction, we demonstrate that matching the sampling matrix in blanks significantly reduced background signals, refining lipid profiles to retain ones likely to be intrinsic to single cells. Specifically, the use of both mobile phase and sampling media to make up capillary blanks provided a more representative baseline, effectively subtracting background noise and ensuring that the lipids identified were not contaminants introduced during sampling, sample transfer or MS analysis. This observation emphasises that the accuracy of single-cell data improves as the blank correction becomes more representative of the sampling conditions. Thus, establishing a blank correction protocol tailored to each experimental setup is essential for achieving precise single-cell lipidomic profiles, allowing for more reliable data interpretation in single-cell analyses.

The findings presented here are particularly relevant for untargeted lipidomics and discovery-based studies, where broader lipid coverage and reduced background interference are critical. For targeted analyses, more stringent criteria, such as higher signal-to-background thresholds, may be required to enhance specificity. This distinction underscores the need to tailor data curation strategies to the study's objectives.

While blank correction minimised non-cellular signals, it remains a challenging aspect of single-cell analysis. Variability in the co-aspiration of media alongside single cells makes it difficult to accurately replicate sampling conditions in blanks. Furthermore, the microenvironment surrounding cells is highly complex and includes factors such as local lipid exchange, which is a dynamic process and therefore may not fully reflect averaged blank media. Even the cell culture media, although free from FBS, contains lipids that can confound results. Consequently, there is a risk of overcorrection when applying stringent blank subtraction. Despite these challenges, our workflow retained a significant number of lipids, highlighting its practicality for single-cell analysis.

### Capillary tip selection can impact the consistency and intensity of lipid profiles in single cells

To evaluate the influence of capillary tip brands (Yokogawa and Humanix) on single cell lipid detection, we first used Humanix tips and performed the three steps of blank correction (no correction, solvent blank + 3 × *S*/*B*, and capillary blank + 3 × *S*/*B*) as discussed in the previous section. Similar results as seen for the Yokogawa tips were observed for the Humanix tips (ESI Fig. 4†). Thus, capillary blank correction was chosen for the data in the following sections.

To compare Yokogawa and Humanix tips, only manual capillary sampling was performed, keeping every other parameter constant. Fig. 3a reveals no statistically-significant difference in the average number of lipid features detected in single cells between Humanix (*n* = 9) and Yokogawa tips (*n* = 10), with an average of 150 lipids detected with Humanix and 140 lipids detected with Yokogawa tips. A second batch of cells, for the same experiment measured on a different day confirmed this result (ESI Fig. 5†). However, PCA (ESI Fig. 6†), highlighted a separation between the data collected from the two tip types, and a Hotelling's *T*^2^ test (ESI Fig. 7†) confirmed this observation (*p* < 3.2 × 10^−8^). Fig. 3b illustrates the number of common (98) and unique lipids found with 100% consistency in single cells for each tip type (19 lipids unique for Yokogawa and 23 for Humanix tips), with a 70% overlap in lipids found with both tip types. However, over half of these lipids had significantly different intensities between tip brands, with most of these lipids (84%) showing higher intensities in cells picked with Yokogawa tips. Additionally, Fig. 3c demonstrates that Yokogawa tips gave greater consistency in lipid detection between single cells compared to Humanix tips, with 128 lipids being detected 100% of the time using Yokogawa tips compared to 98 lipids using Humanix tips.

Consequently, the selection of capillary tip type can play a significant role in the consistency and intensity of lipid detection in single-cell lipidomics workflows. Variations in manufacturing quality can affect both the detected lipid profiles and their variability.^41^ In this study, comparative analysis highlighted that the Yokogawa tips showed greater performance across samples, reflected in more reliable detection rate and higher reproducibility of lipid species. This highlights the value of evaluating different components of single-cell workflows, especially as specialised equipment can be expensive. In this case, employing capillary tips that minimise variability is critical, as it supports replicability and reproducibility in a field where natural sample heterogeneity makes these goals challenging.

### Comparison of automated and manual capillary sampling on lipid profiles in single cells

We next compared automated and manual capillary sampling, keeping all other variables constant and only using Yokogawa tips. As shown in Fig. 4a, the average number of lipid features detected per cell (*n* = 10 per condition) was similar between the two sampling methods (*p* = 0.62), with 138 lipids detected on average with automated and 140 lipids with manual capillary sampling. Similarly, the average number of lipid features between individual lipid classes for both methods were not statistically different (ESI Fig. 10†). We then assessed the total peak area for detected lipids to examine potential differences in signal intensity. As indicated in Fig. 4b, a two-tailed *t*-test comparing the sum of peak areas per cell between automated and manual sampling (*n* = 10 each) revealed no statistically-significant difference (*p* = 0.27), with an average intensity of 7.3 × 10^8^ and 7.1 × 10^8^ for the methods respectively.

For further analysis, tentative lipid identifications were matched with a retention time and polarity-based machine learning algorithm,^37^ to exclude outliers, and filtered to include only lipids from an in-house lipid database (see Experimental section). Additionally, only lipids detected with 100% consistency across each sampling method were included (*n* = 10 for automated and manual single-cell samples). We conducted a Mann–Whitney *U* test for each lipid (ESI Table 1†), which showed that 92% of lipids did not show statistically-significant differences in intensity between the two sampling approaches.

The high similarity in lipid fingerprints obtained from both automated and manual capillary sampling is encouraging. This suggests that each method can capture the cellular lipidome reproducibly, provided that appropriate blank correction is applied. It also demonstrates that automated sampling, with its advantage of ease of use relative to manual sampling, can be effectively deployed in high-throughput studies, while manual sampling may offer flexibility in sampling cells from unconventional surfaces other than imaging culture dishes, albeit with lower sampling throughput.

### Effect of sampling medium and oversampling on sensitivity and lipid profile in single cells

Our final comparison aimed to determine whether the choice of sampling medium—phosphate-buffered saline (PBS) or foetal-bovine serum (FBS)-free cell culture media—impacts lipid profiles when using either automated or manual capillary sampling. We sampled single cells from the same cell line and passage number in either PBS or FBS-free media on the same day. We also performed intentional oversampling of PBS along with single cells to evaluate whether excess medium aspirated during sampling could influence lipid detection sensitivity. Blank and 3 × signal-to-background correction were conducted using capillary blanks containing mobile phase, internal standard, and either PBS or FBS-free media matching the sampling medium used during capillary sampling.

**Fig. 5:**
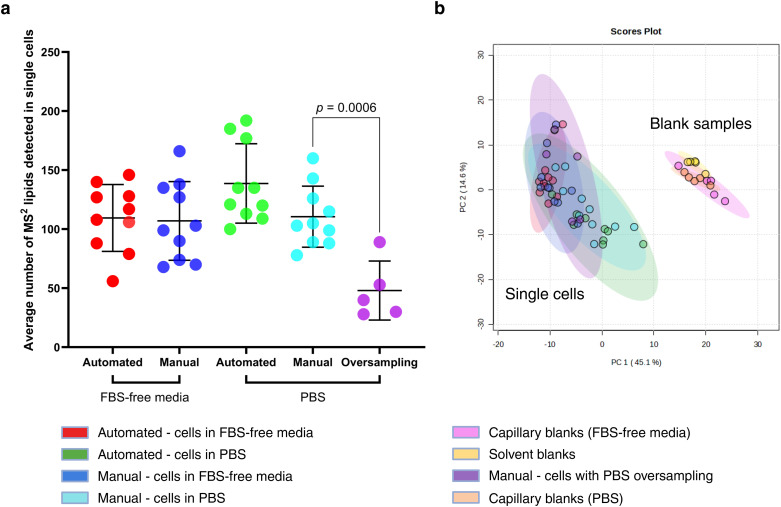
Comparing sampling media in automated and manual capillary sampling. (a) Average number of MS/MS lipids detected per single cell captured with automated and manual capillary sampling using phosphate-buffered saline (*n* = 10 for both sampling methods) and foetal-bovine serum-free media (*n* = 10 for both sampling methods), with additional samples where the PBS volume was purposefully oversampled (*n* = 5). Data are blank corrected with PBS-containing capillary blanks for cells sampled in PBS and FBS-free media capillary blanks for cells sampled in FBS-free media. Error bars show 1 × standard deviation, *p* = 0.0006, (b) PCA of MS/MS lipids from capillary-sampled single cells and blanks. Showing non-blank corrected data from single cells picked in PBS (*n* = 10 for automated and manual method) and FBS-free media (*n* = 10 for automated and manual method), with extra samples (*n* = 5) where PBS was deliberately oversampled. Also showing capillary blanks with corresponding media (PBS or FBS-free media) and solvent blanks (*n* = 5 per group). Data were log transformed and auto scaled for multivariate analysis.

Lipid profiles in single cells remained consistent regardless of whether PBS or FBS-free media was used, as shown in Fig. 5a. However, oversampling PBS significantly reduced the average number of detected lipids in single cells (*p* = 0.0006) compared to FBS-free media. The average number of lipid features detected did not significantly differ between automated and manual capillary sampling, consistent with our previously discussed findings (Fig. 4a).

Principal component analysis (PCA) of non-blank-corrected data (Fig. 5b) supported these findings: single-cell lipid profiles clustered closely, regardless of the sampling medium, suggesting stable lipid profiles across sampling media. In contrast, blanks (capillary blanks with either sampling medium, and solvent blanks) clustered separately from single cells. ESI Fig. 8† further illustrates the clear separation of capillary blanks from solvent blanks by PCA.

Our exploration of the effect of sampling media on lipid detection confirmed the consistency of lipid profiles across different sampling media, as both PBS and FBS-free media yielded consistent lipid profiles. The similarity across media choices highlights the versatility of capillary sampling, enabling researchers to adapt the method to whichever medium of the two is most fit for purpose without introducing substantial artefacts.^42^ However, the observed decrease in lipid detection sensitivity with deliberate PBS oversampling highlights the need for controlled aspiration volumes to minimise ionisation suppression effects and preserve analytical sensitivity. This finding builds on literature indicating that excess sampling medium can dilute or mask cellular signals, suggesting that precise volume control is essential in single-cell studies where detection sensitivity is paramount.^43^

## Conclusions

Overall, these insights underscore the adaptability of capillary sampling for single-cell lipidomics, with both automated and manual techniques supporting reliable lipid profiling. Researchers aiming to capture metadata, such as spatial coordinates, changes in live cell cultures over time, or the effect of cell–cell and cell–matrix interactions, have two viable sampling options to achieve these goals.

While we could not detect a difference in manual and automated sampling of single cells, the similarity in results may be constrained by current LC-MS sensitivity, which limits the discrimination power. In future, more sensitive approaches may be able to detect differences arising from the lack of controlled environmental conditions in manual sampling. Additionally, the complexity of cellular microenvironments, including dynamic lipid exchanges and secretions within cell cultures, cannot be fully replicated in blanks. Developing blank correction methods that better account for these factors may prove helpful in advancing single-cell lipidomics. Future work should also explore improvements in sampling techniques, such as better control of co-aspirated volumes, and leverage advancements in mass spectrometry technology to enhance detection capabilities.

This study represents a significant step forward in single-cell lipidomics by establishing best practices for capillary sampling and addressing key challenges in lipid detection, blank correction, and sampling standardisation. By demonstrating that automated and manual capillary sampling yield comparable results when key parameters are controlled, we provide a robust and adaptable workflow that enhances reproducibility in single-cell lipidomics. The insights gained here lay a foundation for more robust lipidomics analyses, enabling researchers to better explore cellular heterogeneity, identify biomarkers, and advance disease research. As single-cell lipidomics continues to evolve, the methodologies outlined in this work will be instrumental in refining protocols and expanding the applications of this powerful technique in biomedical and metabolic research.

## Author contributions

A.K and M.J.B. conceptualised the study. A.K. performed experiments and data analysis with input from J. v G., M.S., and K.S. Single-cell sampling using the SS2000 by Yokogawa was done by E.F. Lipid retention time correction was performed using an algorithm written by M.S. Composition of the original manuscript was done by A.K. All authors reviewed, edited, and approved the final version of the manuscript.

## Data availability

Data for this article, including .raw and .wiff files are available at Zenodo repository at (https://zenodo.org/records/14162175). All scripts are available on GitHub (https://github.com/AnastasiaKontiza/Capillary_sampling_paper).

## Conflicts of interest

There are no conflicts to declare.

## Supplementary Material

AN-150-D5AN00037H-s001
