# Supplementary material for: Single-cell lipidomics: protocol development for reliable cellular profiling using capillary sampling
Source: Analyst. 2025 Mar 7;150(7):1261–70. doi: 10.1039/d5an00037h (PMC11886952; doi:10.1039/d5an00037h)
Supplement: AN-150-D5AN00037H-s001 [file AN-150-D5AN00037H-s001.pdf]

Supplementary Information (SI) for Analyst.
This journal is © The Royal Society of Chemistry 2025

Supplementary Information

Single-Cell Lipidomics: Protocol Development for Reliable Cellular Profiling using Capillary Sampling

Anastasia Kontiza,[a] Johanna von Gerichten,[a] Matt Spick,[b] Emily Fraser,[a] Catia Costa,[c] Kyle D. G. Saunders,[a] Anthony D. Whetton,[d] Carla F. Newman[e] and Melanie J. Bailey[a,*]

[a]School of Chemistry and Chemical Engineering, Faculty of Engineering and Physical Sciences, University of Surrey, GU2 7XH Guildford, UK

[b]School of Health Sciences, Faculty of Health and Medical Sciences, University of Surrey, GU2 7XH Guildford, UK

[c]School of Computer Science and Electronic Engineering, Faculty of Engineering and Physical Sciences, University of Surrey, GU2 7XH Guildford, UK

[d]vHive, School of Veterinary Medicine, School of Biosciences and Medicine, University of Surrey, Guildford, GU2 7XH, UK

[e]GlaxoSmithKline, Cellular Imaging and Dynamics – Stevenage, SG1 2NY, UK

*m.bailey@surrey.ac.uk

Contents:

Supplementary Figures 1-10

Supplementary Tables 1-3

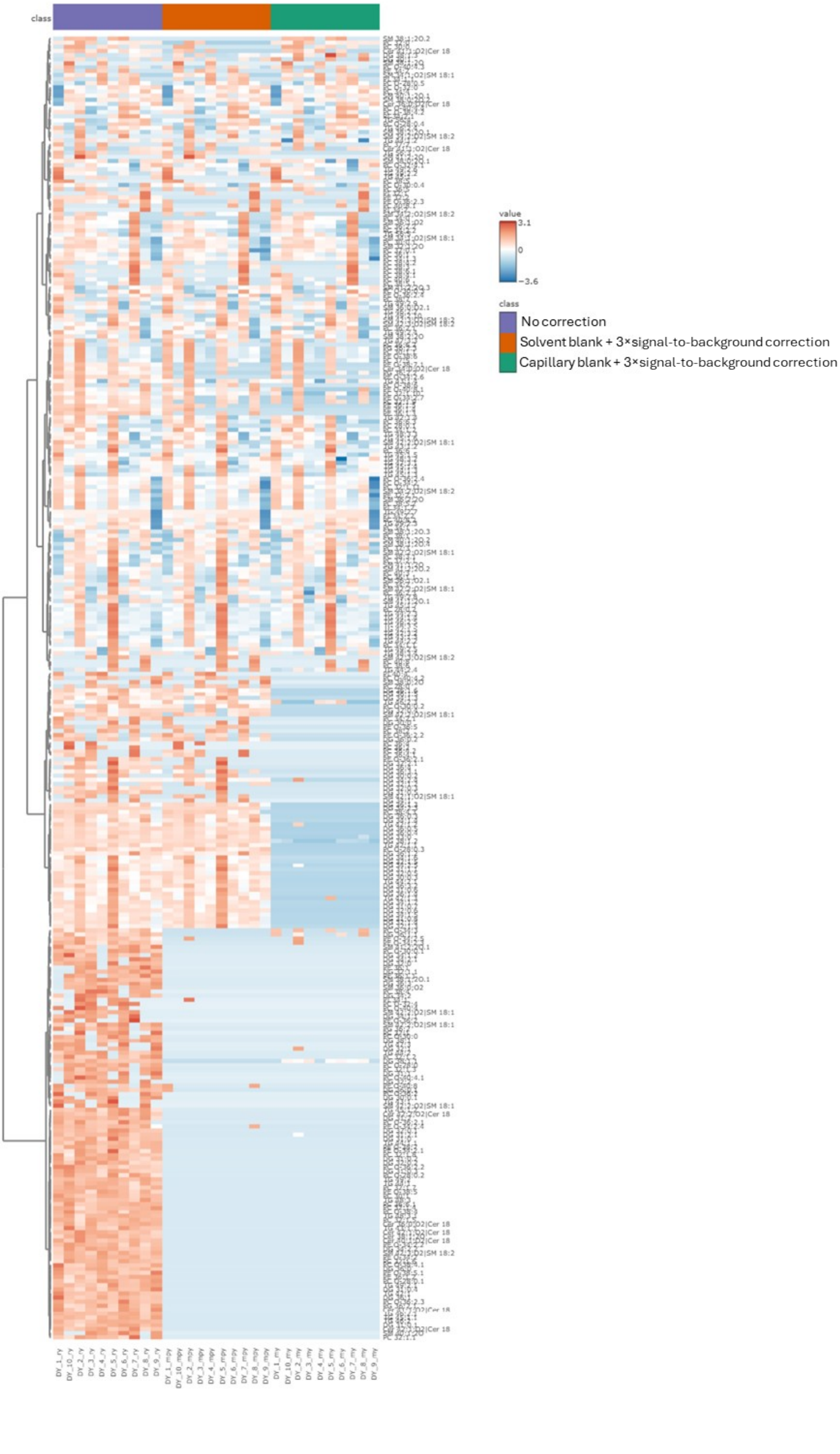
class
value
3.1
0
−3.6
class
No correction
Solvent blank + 3×signal-to-background correction
Capillary blank + 3×signal-to-background correction

Fig. 1 | Effect of blank correction on single-cell data – heatmap with lipids included in y axis. Full clustered heatmap of lipidomics single cells collected using manual capillary sampling with Yokogawa tips; Purple = no blank correction, Orange = solvent blank and 3×signal-to-background correction, Green = capillary blank and 3×signal-to-background correction.

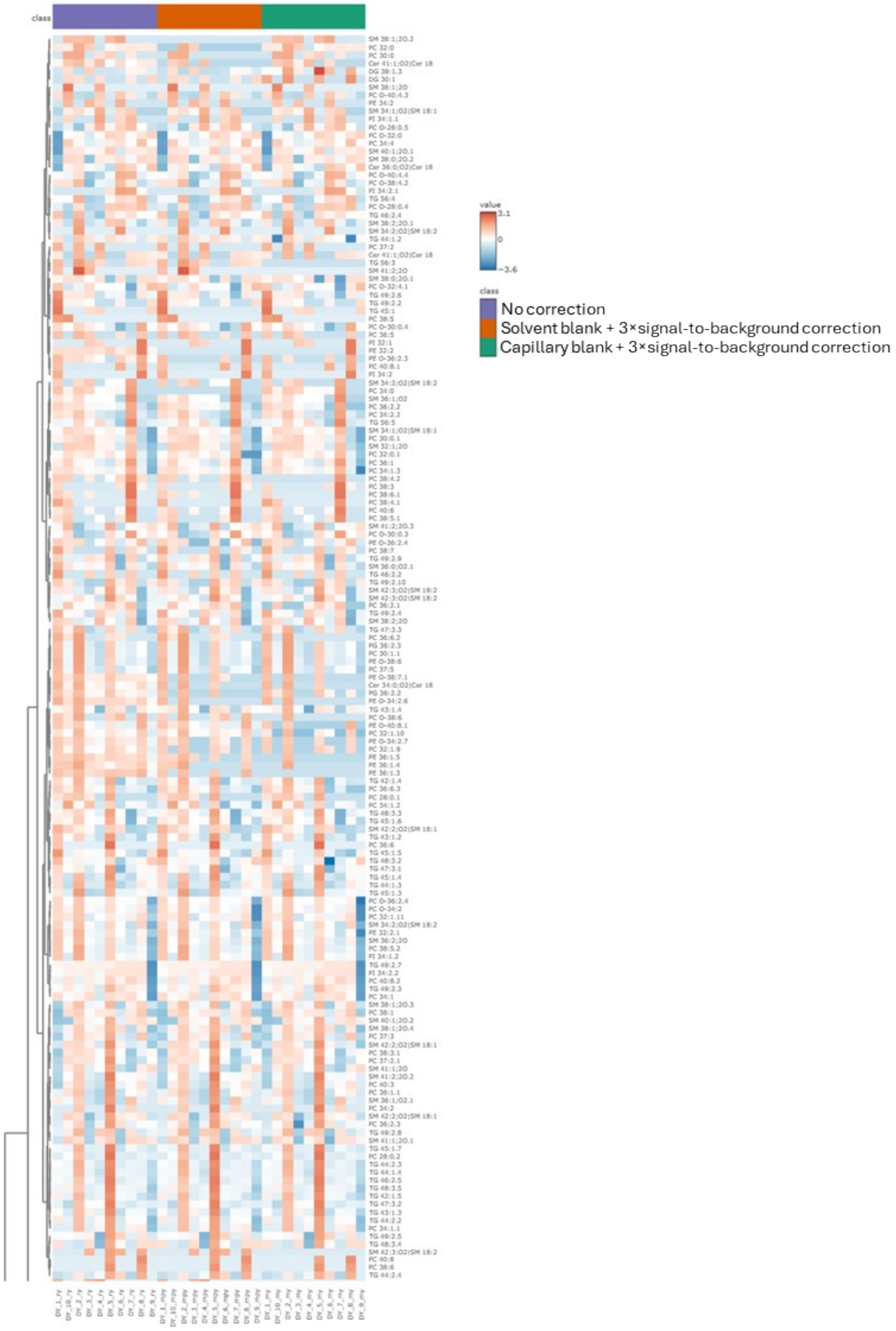

Fig. 2 | Effect of blank correction on single-cell data – heatmap with lipids included in y axis. Top half of clustered heatmap of lipidomics single cells collected using manual capillary sampling with Yokogawa tips; Purple = no blank correction, Orange = solvent blank and 3×signal-to-background correction, Green = capillary blank and 3×signal-to-background correction.

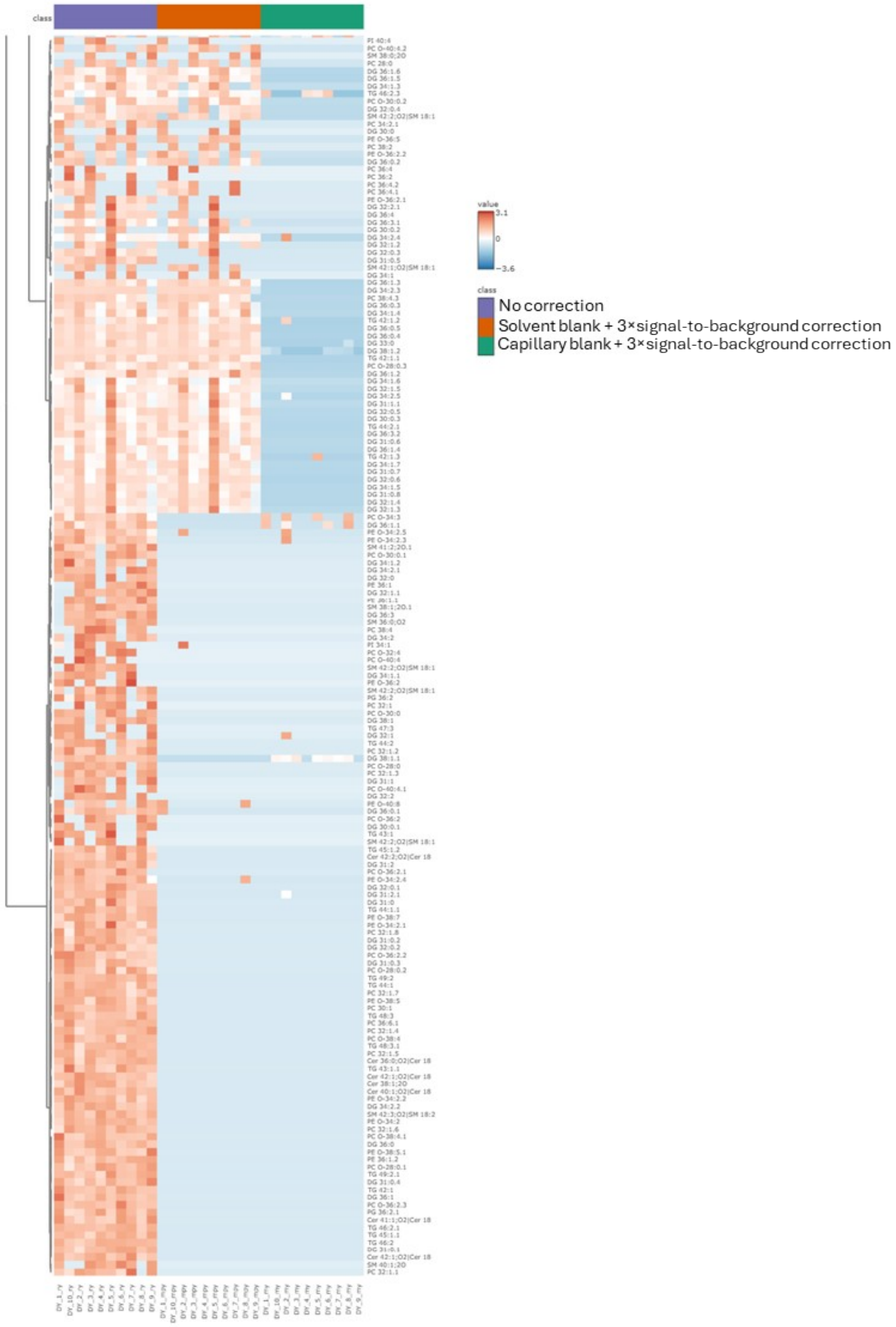

Fig. 3 | Effect of blank correction on single-cell data – heatmap with lipids included in y axis. Bottom half of clustered heatmap of lipidomics single cells collected using manual capillary sampling with Yokogawa tips; Purple = no blank correction, Orange = solvent blank and 3×signal-to-background correction, Green = capillary blank and 3×signal-to-background correction.

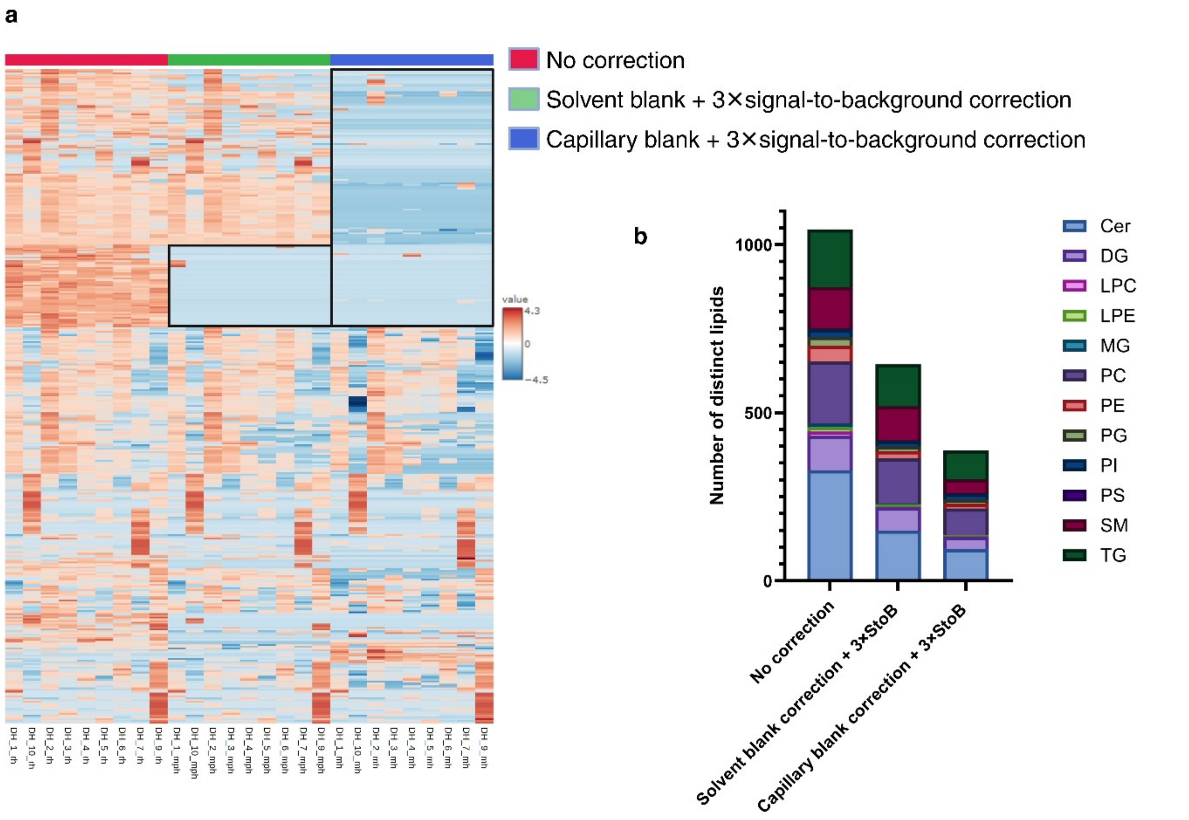

Fig. 4 | Effect of blank correction on single-cell data. a Clustered heatmap of lipidomics single cells collected using manual capillary sampling with Humanix tips; Red = no blank correction, Green = blank and 3×signal-to-background correction using solvent blanks, Blue = blank and 3×signal-to-background correction using capillary blanks (FBS-free media), **b** Stacked bar chart of lipids identified in single cells by lipid class. From left to right, data not corrected, solvent blank and 3×signal-to-background correction, and lastly capillary blank and 3×signal-to-background correction. Only lipids that are present in at least 50% of single cells are shown. Lipidomics identifications were verified with a retention time and polarity-based machine learning algorithm.

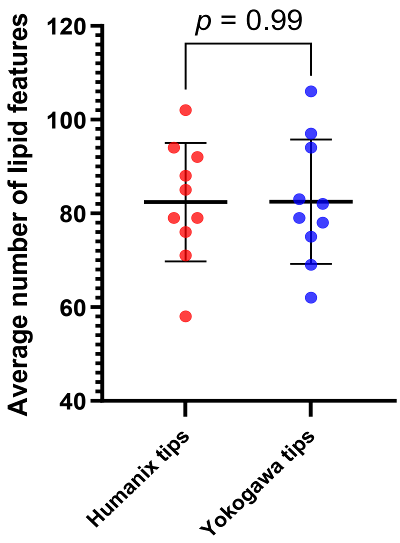

Fig. 5 | Capillary tip effect on lipid signatures – repeat experiment. a Average number of MS1 lipid features detected per single cell sampled with manual capillary sampling using Humanix tips (n=10) and Yokogawa tips (n=10), error bars show 1×standard deviation, *p* = 0.99. Lipidomics identifications were verified with a retention time and polarity-based machine learning algorithm, as well as filtered to include only lipids belonging to a previously-observed lipid database.

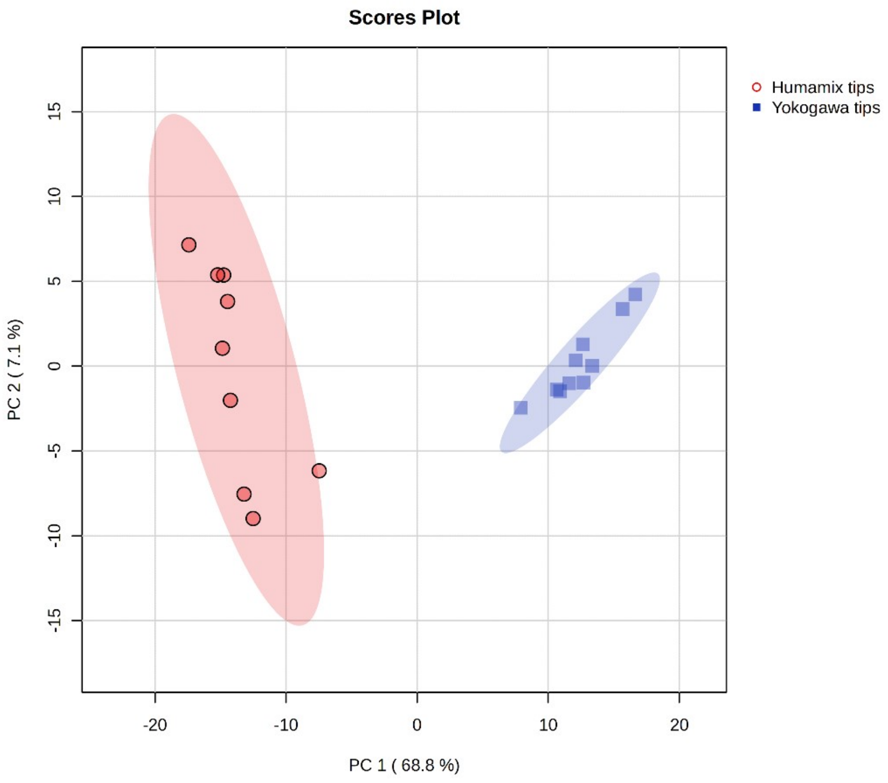

Fig. 6 | Exploring the difference between capillary tip types. PCA of the lipid profiles detected in single cells sampled using Humanix versus Yokogawa tips. N=10 for Yokogawa tips and N=9 for Humanix tips. Data are auto scaled and log

transformed. Lipidomics identifications were verified with a retention time and polarity-based machine learning algorithm, as well as filtered to include only lipids belonging to a previously-observed lipid database.

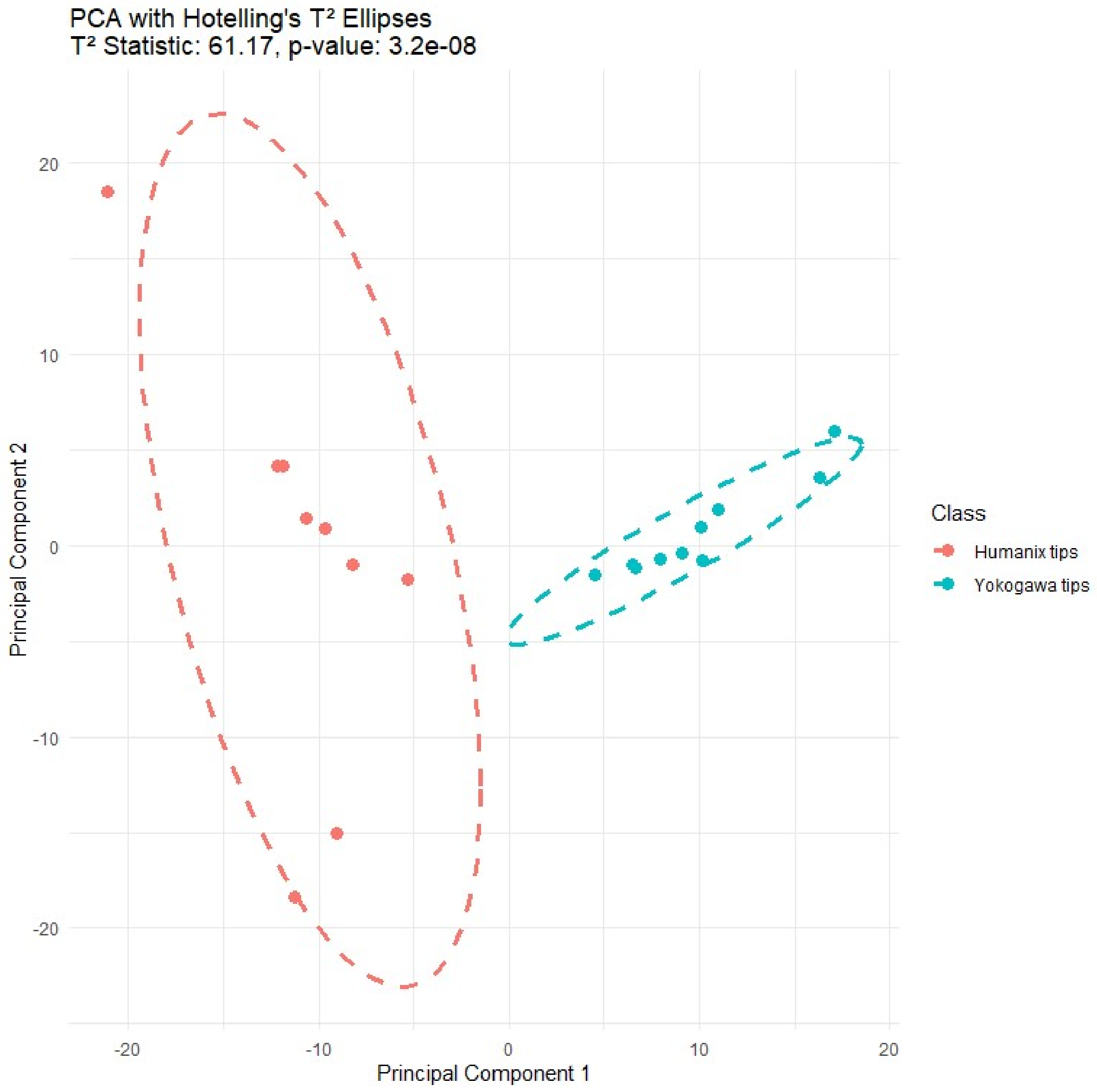

Fig. 7 | Exploring the difference between capillary tip types. Hotelling's T^2 test with PCA reduction between Yokogawa (n=10) and Humanix (n=9) tips. Data are log transformed, and auto scaled. Lipidomics identifications were verified with a retention time and polarity-based machine learning algorithm, as well as filtered to include only lipids belonging to a previously-observed lipid database.

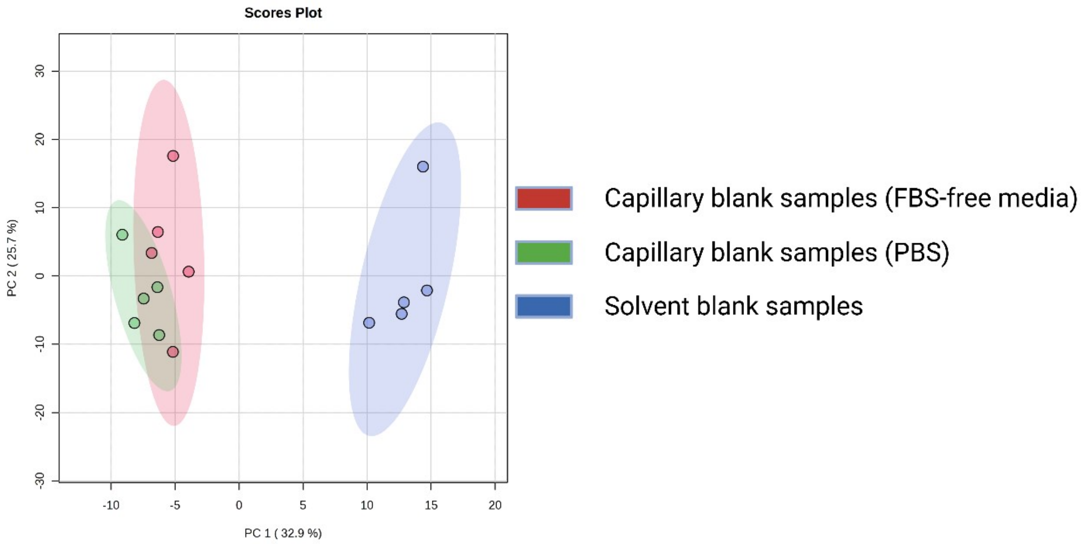

Fig. 8 | Varying sampling medium in capillary sampling – a look at the blanks. PCA of lipids in capillary blanks (PBS and FBS-free media) and solvent blanks. Data are log transformed and auto scaled.

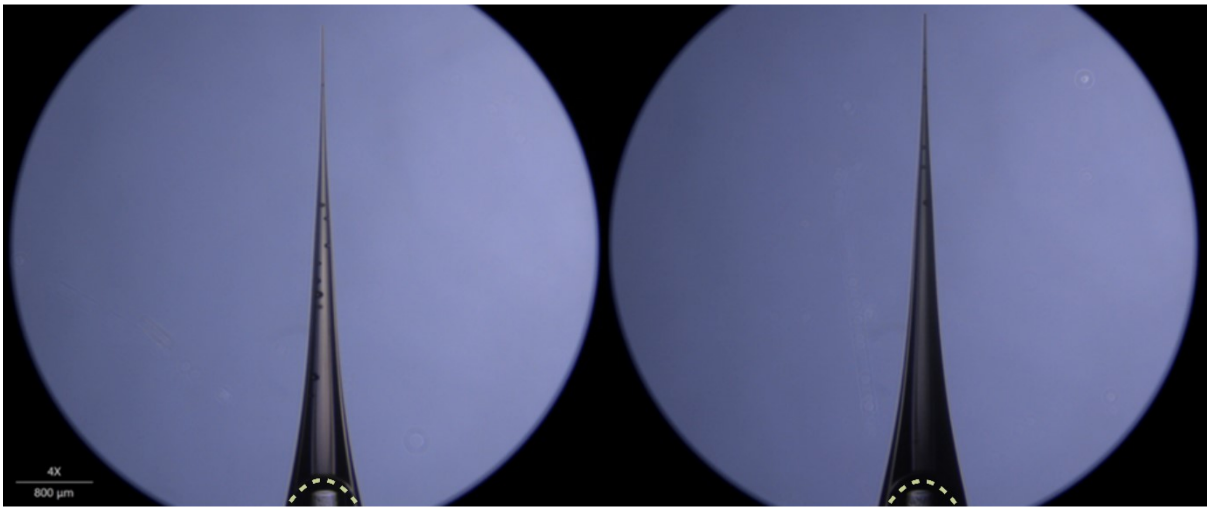

Fig. 9 | Creating capillary blanks. Volume of medium (FBS-free culture media) in Yokogawa capillary tip with cell (left) and capillary blank with medium (FBS-free media) in capillary tip (right). Dotted lines indicate meniscus of medium volume aspirated during capillary sampling.

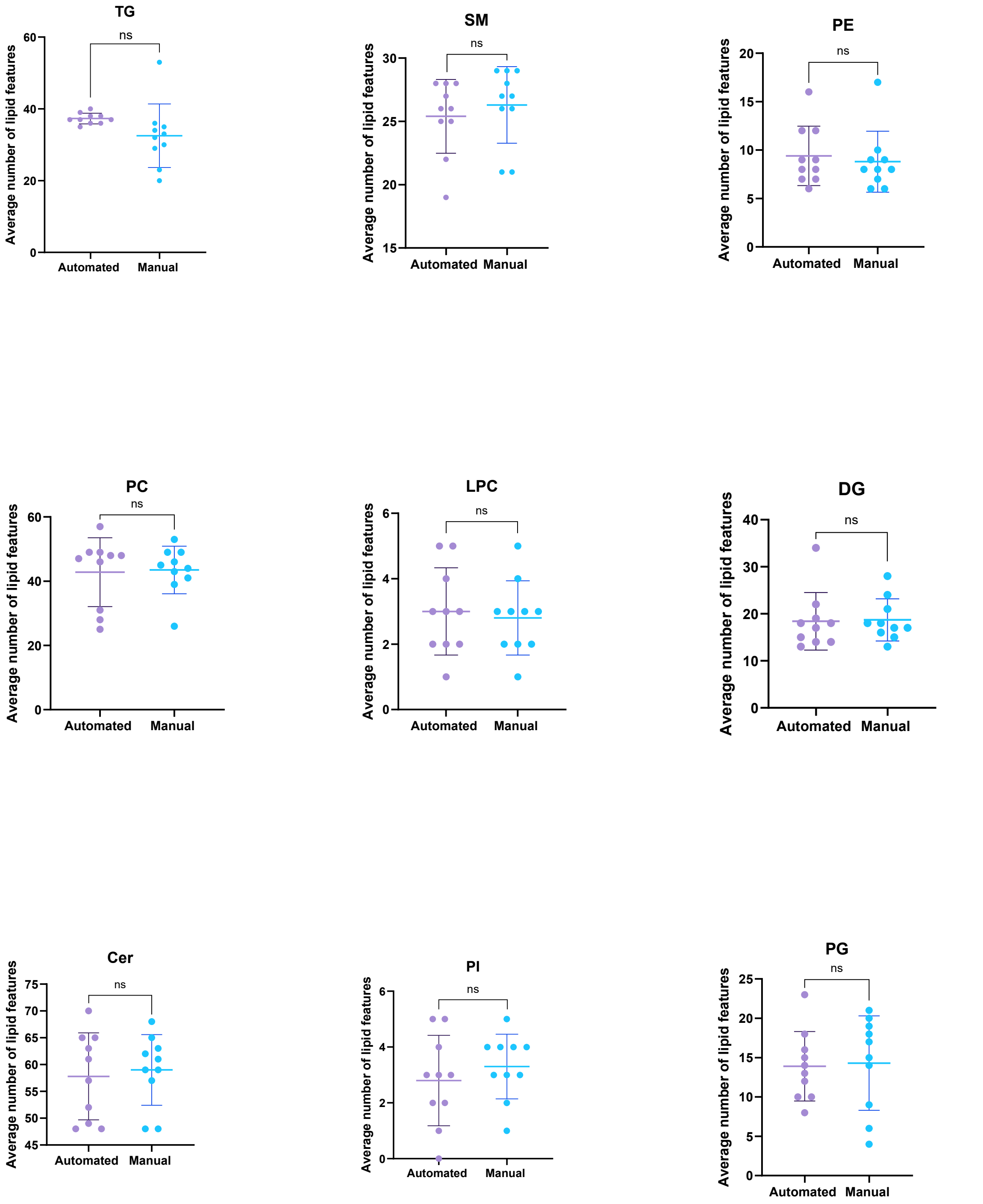

Fig. 10 | Lipid class detection in manual and automated capillary sampling. Average number of MS1 lipid features per class detected in each single cell sampled with manual (blue) and automated (purple) capillary sampling, error bars show 1×standard deviation. Lipidomics identifications were verified with a retention time and polarity-based machine learning algorithm, as well as filtered to include only lipids belonging to a previously-observed lipid database.

Table 1. | Comparison of manual and automated capillary sampling. Results of Mann-Whitney U test on high-confidence lipid intensities detected between automated and manual capillary sampled single cells.

Metabolite name	U statistic	p-value
Cer 36:0;O2|Cer 18:0;O2/18:0	84	0.01133
PC 28:0	57	0.623176
PC 30:0	56	0.677585
PC 30:1	50	1
PC 32:0	51	0.96985
PC 32:1	47	0.850107
PC 34:0	48	0.909722
PC 34:1	47	0.850107
PC 34:1	66	0.241322
PC 34:2	40	0.472676
PC 34:3	57	0.623176
PC 34:4	57	0.623176
PC 35:1	66	0.241322
PC 36:1	58.5	0.545199
PC 36:2	59	0.520523
PC 36:3	66	0.241322
PC 37:5	52	0.909722
PC 38:1	61	0.427355
PC 38:3	66	0.241322
PC 38:5	48	0.909722
PC 40:3	35	0.273036
PC 40:8	35	0.273036
PC O-28:0	82	0.017257
PC O-30:0	53	0.850107
PC O-30:1	60	0.472676
PC O-32:0	34	0.241322
PC O-32:4	49	0.96985
PC O-34:2	37	0.344704
PC O-36:2	37	0.344704
PC O-38:4	47	0.850107
PC O-40:4	50	1
PE 32:2	32	0.185877
PE O-38:6	56	0.677585
PG 36:2	50	1
PI 34:1	39	0.427355
PI 34:2	27	0.088973
SM 32:1;2O	40	0.472676
SM 34:1;O2|SM 18:1;O2/16:0	47	0.850107
SM 34:2;O2|SM 18:2;O2/16:0	35	0.273036
SM 36:0;O2	72	0.10411
SM 36:2;2O	38	0.384673
SM 38:0;2O	85	0.009108
SM 34:0;2O	81	0.021134

**SM 38:1;2O**	57	0.623176
**PC 37:3**	66	0.241322
**SM 38:2;2O**	41	0.520523
**SM 40:1;2O**	73	0.088973
**PC 35:2**	44	0.677585
**SM 41:1;2O**	67	0.212294
**PC 35:3**	74	0.075662
**SM 41:2;2O**	65	0.273036
**PC 38:2**	64	0.307489
**SM 42:2;O2|SM 18:1;O2/24:1**	61	0.427355
**SM 42:3;O2|SM 18:2;O2/24:1**	51	0.96985
**SM 42:3;O2|SM 18:2;O2/24:1**	57	0.623176
**TG 42:1**	30	0.140465
**TG 43:1**	23	0.045155
**DG 36:2**	43	0.623176
**TG 44:1**	34	0.241322
**DG 32:0**	56	0.677585
**TG 44:2**	33	0.212294
**DG 36:0**	29	0.121225
**TG 45:1**	44	0.677585
**DG 34:0**	43	0.623176
**DG 38:1**	70	0.140465
**DG 36:1**	43	0.623176
**TG 46:2**	27	0.088973
**TG 47:3**	31	0.161972
**TG 48:3**	17	0.014019
**TG 44:2**	40	0.472676
**TG 45:1**	27	0.088973
**TG 46:2**	49	0.96985
**TG 46:3**	25	0.064022
**TG 47:3**	50	1
**TG 48:3**	33	0.212294
**TG 49:2**	48	0.909722
**TG 56:3**	45	0.73373
**TG 56:4**	76	0.053903

Table 2. | Liquid chromatography gradient used in LC-MS experiments. Mobile Phase A 60:40 (v/v) acetonitrile/water and mobile Phase B 85:10:5 (v/v) isopropanol/water/acetonitrile, both containing 0.1 % (v/v) formic acid and 10 mM ammonium formate; flow rate of 0.35 mL/min.

Time (min)	% A	% B
0.0	70	30
5.0	70	30
5.1	57	43
14	30	70
14.1	30	70

21	1	99
24	1	99
24.1	70	30
28	70	30

Table 3. | Liquid chromatography gradient used in LC-MS/MS experiments. Mobile Phase A 60:40 (v/v) acetonitrile/water and mobile Phase B 85:10:5 (v/v) isopropanol/water/acetonitrile, both containing 0.1 % (v/v) formic acid and 10 mM ammonium formate; flow rate of 8 μL/min.

Time (min)	% A	% B
0.0	60	40
0.5	60	40
4.5	1	99
6.5	1	99
6.5	20	80
11	60	40
15	60	40
